# Contrasting effect of the latency-reversing agents bryostatin-1 and JQ1 on astrocyte-mediated neuroinflammation and brain neutrophil invasion

**DOI:** 10.1186/s12974-017-1019-y

**Published:** 2017-12-11

**Authors:** Alizé Proust, Corinne Barat, Mathieu Leboeuf, Jean Drouin, Michel J. Tremblay

**Affiliations:** 1 Axe des Maladies Infectieuses et Immunitaires, Centre de Recherche du CHU de Québec-Université Laval, Pavillon CHUL, Québec, G1V 4G2 Canada; 2 Département d’obstétrique, gynécologie et reproduction, Faculté de Médecine,, Université Laval, Québec, G1V 0A6 Canada; 3 Département de médecine familiale et d’urgence, Faculté de Médecine, Université Laval, Québec, G1V 0A6 Canada; 4 Département de Microbiologie-Infectiologie et Immunologie, Faculté de Médecine, Université Laval, Québec, G1V 0A6 Canada

**Keywords:** CNS, Astrocytes, LRA, HIV-1 latency, Neuroinflammation, Excitotoxicity, NETosis

## Abstract

**Background:**

Despite effectiveness of the combined antiretroviral therapy, HIV-1 persists in long-lived latently infected cells. Consequently, new therapeutic approaches aimed at eliminating this latent reservoir are currently being developed. A “shock and kill” strategy using latency-reversing agents (LRA) to reactivate HIV-1 has been proposed. However, the impact of LRA on the central nervous system (CNS) remains elusive.

**Methods:**

We used human fetal astrocytes and investigated the effects of several LRA on their functional and secretory activities. Astrocytes were infected with VSV-G-pseudotyped HIV-1 before treatment with various blood-brain barrier (BBB)-permeable LRA at subcytotoxic doses, which allow HIV-1 reactivation based on previous in vitro and clinical studies. Cells and supernatants were then used to evaluate effects of infection and LRA on (i) viability and metabolic activity of astrocytes using a colorimetric MTS assay; (ii) chemokines and proinflammatory cytokines secretion and gene expression by astrocytes using ELISA and RT-qPCR, respectively; (iii) expression of complement component 3 (C3), a proxy for astrogliosis, by RT-qPCR; (iv) glutamate uptake capacity by a fluorometric assay; and (v) modulation of neutrophil transmigration across an in vitro BBB model.

**Results:**

We demonstrate that bryostatin-1 induces secretion of chemokines CCL2 and IL-8 and proinflammatory cytokines IL-6 and GM-CSF, whereas their production is repressed by JQ1. Bryostatin-1 also increases expression of complement component 3 and perturbs astrocyte glutamate homeostasis. Lastly, bryostatin-1 enhances transmigration of neutrophils across an in vitro blood-brain barrier model and induces formation of neutrophil extracellular traps.

**Conclusions:**

These observations highlight the need to carefully assess the potential harmful effect to the CNS when selecting LRA for HIV-1 reactivation strategies.

**Electronic supplementary material:**

The online version of this article (10.1186/s12974-017-1019-y) contains supplementary material, which is available to authorized users.

## Background

It has been established that HIV-1 crosses the blood-brain barrier (BBB) and enters the brain in the early stages of infection, which confers to the virus protection from the immune system and certain antiretroviral drugs [1]. While the existing combined antiretroviral therapy (cART) has considerably improved the patients’ quality of life and prevented the progression of the disease and the occurrence of the most severe forms of HIV-1-associated neurocognitive disorders (HAND), the incidence of the mildest forms of neuropathologic abnormalities has actually increased. Moreover, cART fails to completely eliminate HIV-1 in reservoirs and viremia rebounds upon treatment interruption due to the reactivation of latent HIV-1.

The so-called "shock and kill" approach was proposed to eliminate latently infected cells persisting despite long-term effective cART [2]. In this strategy, latent HIV-1 would be reactivated by latency-reversing agents (LRA) (shock) and the infected cells would then be eliminated by the immune system (kill) while cART would protect from new rounds of virus infection. However, none of the current LRA target exclusively latently infected cells, and although their effects have been extensively studied in vitro in the blood compartment [3], the consequences of these treatments in the central nervous system (CNS) are still unclear. Importantly, the outcome of the “shock and kill” approach could be adversely affected by some unique characteristics of the CNS such as (i) constrained LRA penetration which may limit the “shock” [4], (ii) reduced immune surveillance which may compromise the “kill” [5], (iii) extensive virus compartmentalization leading to genetically distinct variants responding differently to LRA [6, 7], and (iv) altered cART bioavailability which may allow sustained viral replication [8]. Our group has previously shown that the LRA bryostatin-1 and prostratin, two protein kinase C (PKC) agonists, caused inflammation and disruption of the BBB, as well as alteration of leukocyte adherence and transmigration [9]. Thus, a careful assessment of the potential impact of LRA on long-lived infected cells of the CNS such as astrocytes is essential to evaluate if the “shock and kill” approach would be appropriate or even viable for the brain reservoir.

The aim of this manuscript is to evaluate the overall safety of different LRA in a tightly regulated microenvironment such as the CNS. For this purpose, we treated human astrocytes with various LRA that can cross the BBB and analyzed their overall effects on some specific cellular functions. Our results demonstrate that bryostatin-1 induces astrogliosis and disturbs the astrocytic glutamate uptake/release balance, which can lead to excitotoxicity. Moreover, bryostatin-1 could induce neuroinflammation since we report that it drives secretion of certain chemotactic factors and proinflammatory cytokines such as CCL2 (also known as monocyte chemoattractant protein-1, MCP-1), interleukin-6 (IL-6), IL-8, and granulocyte-macrophage colony-stimulating factor (GM-CSF) by astrocytes. Using an in vitro BBB model, we show that transmigration of neutrophils is increased in response to bryostatin-1 as well as neutrophil extracellular trap formation. Taken together, these results suggest that bryostatin-1 could induce an inflammatory syndrome in the brain that could eventually lead to neurological disorders.

## Methods

### HIV-1 LRA and positive controls used in this study

BIX-01294 (2-(hexahydro-4-methyl-1*H*-1,4-diazepin-1-yl)-6,7-dimethoxy-*N*-[1-(phenylmethyl)-4-piperidinyl]-4-quinazolinamine trihydrochloride hydrate), SAHA (suberoylanilide hydroxamic acid), HMBA (*N*,*N*′-hexamethylene bis(acetamide)), disulfiram (tetraethylthiuram disulfide), bryostatin-1, and JQ1 were all purchased from Sigma-Aldrich. The positive controls Pam3Csk4 and poly(I:C) were purchased from InvivoGen.

### Virus production

Virions were produced by calcium-phosphate transient transfection in 293T cells as previously described [10]. As astrocytes do not express the primary cell surface receptor for HIV-1 (i.e., CD4), we used VSV-G-pseudotyped HIV-1-based reporter viruses for our infection experiments by co-transfecting 293T with pHCMV-G and NL4-3 eGFP-IRES-Nef *env* [11]. Infectivity of our virus stocks was assessed using the genetically modified HeLa-derived indicator cell line TZM-bl [10].

### Cell culture

The hCMEC/D3 cell line was kindly provided by Dr. Pierre Couraud (Institut Cochin) under the license from the Institut National de la Santé et de la Recherche Médicale (INSERM). This immortalized human BMVEC line possesses the morphological and functional characteristics of cerebral endothelial cells [12] and was maintained in culture as described previously [9]. Briefly, hCMEC/D3 cells were grown in endothelial basal medium-2 (EBM-2; Lonza Group Ltd.) supplemented with 5% fetal bovine serum (Corning Life Sciences), 1% penicillin-streptomycin solution (GIBCO Life Technologies, Invitrogen), 1.4 μM hydrocortisone, 5 μg/mL ascorbic acid, 10 mM HEPES (all three from Sigma-Aldrich), 1% chemically defined lipid concentrate (GIBCO Life Technologies), and 1 ng/mL of basic fibroblast growth factor (ProSpec-Tany Technogene Ltd.). Passage numbers 28-34 were used throughout our experiments.

Neutrophils were purified from human blood samples as described previously [13]. Briefly, blood samples were centrifuged to concentrate the blood and remove platelets before erythrocyte sedimentation in 2% dextran T-500 followed by centrifugation on Ficoll-Paque cushion. Contaminating erythrocytes were removed by hypotonic lysis for 10 s. Neutrophils were resuspended in a medium containing 50% complete EBM-2 and 50% DMEM supplemented with 10% fetal bovine serum.

### Isolation and purification of astrocytes

Human fetal astrocytes were isolated from fetal brain samples (15 to 24 gestational weeks) as previously described [9, 14]. Briefly, blood vessels and meninges were removed from the fetal brain tissues. Thereafter, the tissues were minced and treated with 0.2 mg/mL DNase I (Roche) and 0.25% trypsin (GIBCO Life Technologies) for 30 min before being passed through a 70-μm cell strainer (Corning). The flow through was plated in T75 tissue culture flasks for adherent cells (Sarstedt) at a final concentration of 6–8 × 10^7^ cells/flask in MEM supplemented with 10% FBS, 100 U/mL penicillin, 100 μg/mL streptomycin, 0.3 mg/mL l-glutamine, 1 mM sodium pyruvate, 1× MEM nonessential amino acids, 0.5 μg/mL amphotericin B (all from GIBCO Life Technologies), and 0.1% dextrose (Sigma-Aldrich). Astrocytes were grown at 37 °C under a 5% CO_2_ atmosphere and left untouched for 2 weeks before being passaged once a week. To ensure cell purity, all experiments were conducted on the third or fourth passage.

### Infection with HIV-1 (VSV-G) pseudotypes and treatment with LRA

Astrocytes were seeded in 24-well plates at 2 × 10^5^ cells/well in X-VIVO™ 20 hematopoietic serum-free culture medium (Lonza) and cultured for 24 h before virus infection. Cells were then incubated with VSV-G-pseudotyped HIV-1 (MOI 0.1) for 24 h before being washed thoroughly to remove unbound viral particles and left untouched for six more days to ensure there is no more residual viral activity from the virus inoculum and initial cell responses to acute infection are completed [15]. Cells were then treated with various LRA for 24 h at subcytotoxic doses, which allow HIV-1 reactivation based on previous in vitro and clinical studies.

### MTS assay

The metabolic activity of astrocytes was assessed using CellTiter 96™ AQueous Nonradioactive Cell Proliferation Assay following the manufacturer’s instructions (Promega). Absorbance at 490 nm was measured using an ELX808 microplate reader (Biotek instruments).

### Quantification of mRNA and protein levels

Total RNA was extracted from astrocytes using the RNeasy Kit (Qiagen) according to the manufacturer’s instructions and reverse transcribed to cDNA with M-MLV RT Polymerase (Promega). Cytokine expression was assessed by SYBR-Green Quantitative RT-PCR using QuantStudioTM 6 Flex Real-Time PCR System (Thermo Fisher Scientific) following the manufacturer’s instructions. Primer (used at 0.4 μM) sequences were forward 5′-CCCCAGTCACCTGCTGTTAT-3′ and reverse 5′-TGGAATCCTGAACCCACTTC-3′ for CCL2, forward 5′-TAGCAAAATTGAGGCCAAGG-3′ and reverse 5′-AAACCAAGGCACAGTGGAAC-3′ for IL-8, forward 5′-CCTTCCAAAGATGGCTGAAA-3′ and reverse 5′-CAGGGGTGGTTATTGCATCTC-3′ for IL-6, forward 5′-AAAAGGGGCGCAACAAGTTC-3′ and reverse 5′-GATGCCTTCCGGGTTCTCAA-3′ for C3, and forward 5′-TAGAGGGACAAGTGGCGTTC-3′ and reverse 5′-CGCTGAGCCAGTCAGTGT-3′ for 18S. Amplification of target genes was normalized to the geometric mean of 18S ribosomal cDNA. A standard curve was drawn for each gene of interest using serial dilutions of pooled cDNA from all samples. Levels of CCL2, IL-6, IL-8, GM-CSF, CCL5, and TNF produced by astrocytes were determined by ELISA MAX™ Deluxe assays (Biolegend), and IL-1β concentrations were assessed by ELISA Ready-Set-Go! (eBioscience) following the manufacturer’s instructions. IFNα/β levels were determined using HEK-Blue™ IFN-α/β indicator cells per manufacturer’s instructions (InvivoGen).

### Quantification of TGFβ1

Levels of transforming growth factor beta 1 (TGFβ1) were assessed using the PAI-1/luciferase assay as previously described [16]. Briefly, transfected mink lung epithelial cells (MLEC) kindly provided by Dr. Daniel Rifkin (New York University Medical Center) were plated in a 96-well plate at 1.6 × 10^4^ cells per well in DMEM supplemented with 10% FBS, 100 U/mL penicillin, and 100 μg/mL streptomycin. Thereafter, 100 μL of cell supernatant from astrocytes was added directly to cells. Recombinant TGFβ was used as standards (15.625 to 500 pg/mL; 100 μL/well). Samples and standards were incubated overnight at 37 °C under a 5% CO_2_ atmosphere. Cells were then lysed, and luciferase activity was measured using a Varioskan flash multi-mode reader (Thermo Fisher Scientific).

### Astrocyte glutamate uptake

Glutamate uptake by astrocytes was monitored using the Glutamate assay kit (Abcam). Briefly, mock- and HIV-1-infected astrocytes were either left untreated or treated for 24 h with JQ1, bryostatin-1, or both JQ1 and bryostatin-1 in X-VIVO culture medium. Glutamate levels in the medium were quantified after 2, 4, 8, and 24 h of treatment following the manufacturer’s instructions. A standard curve was drawn using serial dilutions of glutamate.

### In vitro human BBB model system

We used a co-cultivation model including hCMEC/D3 cells and astrocytes seeded on each side of a porous insert allowing cell-cell contacts as previously described [9, 17]. Briefly, cell culture inserts for 24-well plates with 3.0-μm pore translucent PET membrane (Corning Life Sciences) were coated with 150 μg/mL collagen-1 (Sigma-Aldrich). Astrocytes were then seeded on the basal side of the membrane and incubated for 4 h before hCMEC/D3 cells were seeded on the upper side of the membrane. Cells were allowed to grow in 150 μL (upper chamber) and 750 μL (collector) of BBB medium, composed of 50% complete EBM-2 and 50% DMEM supplemented with 10% FBS, during 6 days to reach confluence. BBB integrity was assessed by measuring permeability to dextran-rhodamine. The culture medium in the upper chamber was replaced with BBB medium supplemented with 1 mg/mL 70-kDa dextran-rhodamine (Life Technologies). After 6 h, the fluorescence intensity in collectors was measured using a Varioskan flash multi-mode reader (Thermo Fisher Scientific). Samples displaying dextran-rhodamine permeability superior to 20% of the empty control were discarded.

### Quantification of neutrophil transmigration

BBB-containing inserts were placed into a 24-well plate containing astrocyte-conditioned medium (ACM) before addition of 2 × 10^6^ fleshly isolated neutrophils in the upper chamber. After 4 h of cell transmigration, the plate was centrifuged for 2 min at 100×*g* in order to collect neutrophils bound to astrocytes on the basal side of the insert. Next, inserts were discarded and the absolute number of neutrophils in the collector was counted by flow cytometry using 123count eBeads (eBioscience). In some experiments, neutrophils were pretreated with the noncompetitive allosteric inhibitor of IL-8 reparixin (1 μM) (Cayman Chemical) for 45 min before performing the transmigration assay.

### Microscopy-based detection of NETs

Transmigration assays were performed as described above but using as collector a μ-Plate 24-well ibiTreat (Ibidi) allowing fluorescent microscopy. In parallel, 1 × 10^6^ neutrophils were cultured in the same μ-Plate and remained untreated or treated for 4 h with bryostatin-1 (25 nM) or PMA (100 nM). Thereafter, DNA was labeled with 1× GreenGlo™ Safe DNA Dye (Denville Scientific Inc.) for 30 min at room temperature. Next, neutrophils and NETs were visualized with a fully automated inverted Leica DMI6000 B microscope (Leica Microsystems). Images were acquired using both FITC and UV filter cubes. In some experiments, DNA was labeled with NucBlue® and imaged using a UV filter cube. Image acquisition was done using Volocity Software Version 5.4.0 (PerkinElmer).

### Statistical analysis

Means of raw data were compared using either two-tailed paired Student’s *t* test or one-way ANOVA with the appropriate post-test or the non-parametric Friedman test. *P* values < 0.05 were deemed statistically significant. Calculations were performed with the GraphPad PRISM 7 software for Windows (GraphPad Software).

## Results

### Effective concentrations of LRA are non-toxic for astrocytes

We have included in our study a panel of LRA that are expected to pass through the BBB and are representing the main classes of compounds based on their mode of action (Table 1). We used LRA at concentrations shown to be effective at reversing HIV-1 latency in vitro. However, given the essential role of astrocytes in the BBB structure and function, we also used doses of LRA that are non-toxic for brain microvascular endothelial cells (BMVEC) [9]. We tested various combinations of LRA showing additive or synergistic activities. As an example, PKC agonists were shown to synergize with JQ1 while histone deacetylase (HDAC) inhibitors can induce HIV-1 transcription and virus production [18]. Pam3Csk4 (TLR1/2 agonist) and poly(I:C) (TLR3 agonist) were used as positive controls because of their well-established proinflammatory potential on astrocytes [19, 20].

**Table 1:** Activity, CNS penetrance, and concentrations of LRA and positive controls

Compounds (activity)	CNS penetrance [4]	Concentrations used in our study	HIV-1 reactivating concentrations in vitro	Plasmatic concentrations^a^
Bryostatin-1 (PKC modulator)	++	25 nM	0.25–100 nM [29]	0.05 nM^b^ [28]
JQ1 (BRDi)	++	0.5 μM	0.5 μM [53]	ND
SAHA (HDACi)	+++	1 μM	0.1–10 μM [53–55]	1 μM [56, 57]
HMBA (Tat mimetic)	++	1 mM	1–5 mM [58, 59]	0.0019 mM [60]
Disulfiram (Akt signaller)	+++	1 μM	0.1–10 μM [53, 61, 62]	0.2 μM [63] 2 μM [64]
Bix-01294 (HMTi)	ND	1 μM	1–10 μM [65]	ND
Positive controls: poly(I:C) Pam3Csk4		30 μg/mL 10 μg/mL		

*PKC* protein kinase C, *BRDi* bromodomain inhibitor, *SAHA* suberoylanilide hydroxamide acid, *HDACi* histone deacetylase inhibitor, *HMBA* hexamethylene bisacetamide, *HMTi* histone methyltransferase inhibitor, *TLR* Toll-like receptor, *poly(I:C)* polyinosinic/polycytidylic acid, ++/+++ good/very good penetration, *ND* not determined

^a^Maximum in vivo plasma concentration in humans

^b^No HIV-1 reactivation at this low in vivo plasma concentration in humans

We first investigated proliferation of astrocytes in response to HIV-1 infection and treatment with LRA by measuring their metabolic activity with the colorimetric MTS assay. Infection of astrocytes with VSV-G-pseudotyped HIV-1 diminishes cell metabolic activity (mean decrease of 29.6%) (Additional file 1: Figure S1). However, treatment of astrocytes with LRA had no significant effect on cell viability of both uninfected and virus-infected cells. The modest although statistically significant decrease in metabolic activity seen after JQ1 treatment of uninfected astrocytes was not associated to any morphologic change (data not shown). Thus, it can be concluded that LRA are well tolerated by astrocytes at the tested concentrations.

### Bryostatin-1 induces secretion of chemoattractants and proinflammatory cytokines while JQ1 represses their production

**Fig. 1:**
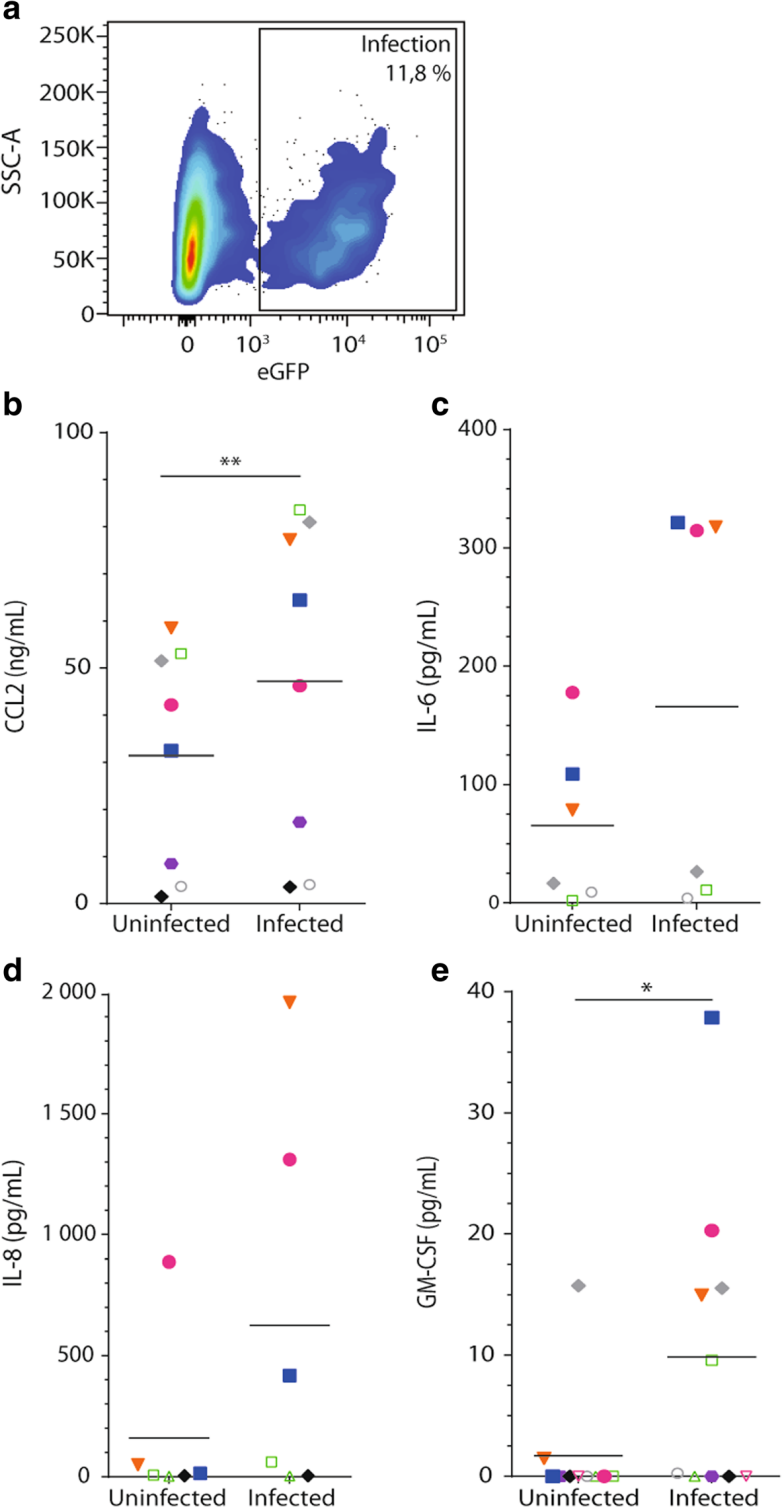
HIV-1 infection promotes secretion of CCL2, IL-6, IL-8, and GM-CSF by astrocytes. Primary human astrocytes were either left uninfected or infected with VSV-G-pseudotyped eGFP-encoding HIV-1 particles for 7 days. **a** Infection was assessed by monitoring the percentage of eGFP-expressing astrocytes by flow cytometry. Data from one representative donor is shown. Next, cell-free supernatants were collected to measure **b** CCL2, **c** IL-6, **d** IL-8, and **e** GM-CSF production by commercial ELISA tests. Results are presented in raw data (i.e., cytokine levels expressed in pg/mL or ng/mL). Data from six to nine experiments with independent donors are shown. Each colored symbol represents a different donor (the same color is preserved for the same donor throughout our study), and the horizontal line represents the grand mean. Asterisks denote statistically significant data as defined by the Student *t* test (*P < 0.05 and **P < 0.01)

**Fig. 2:**
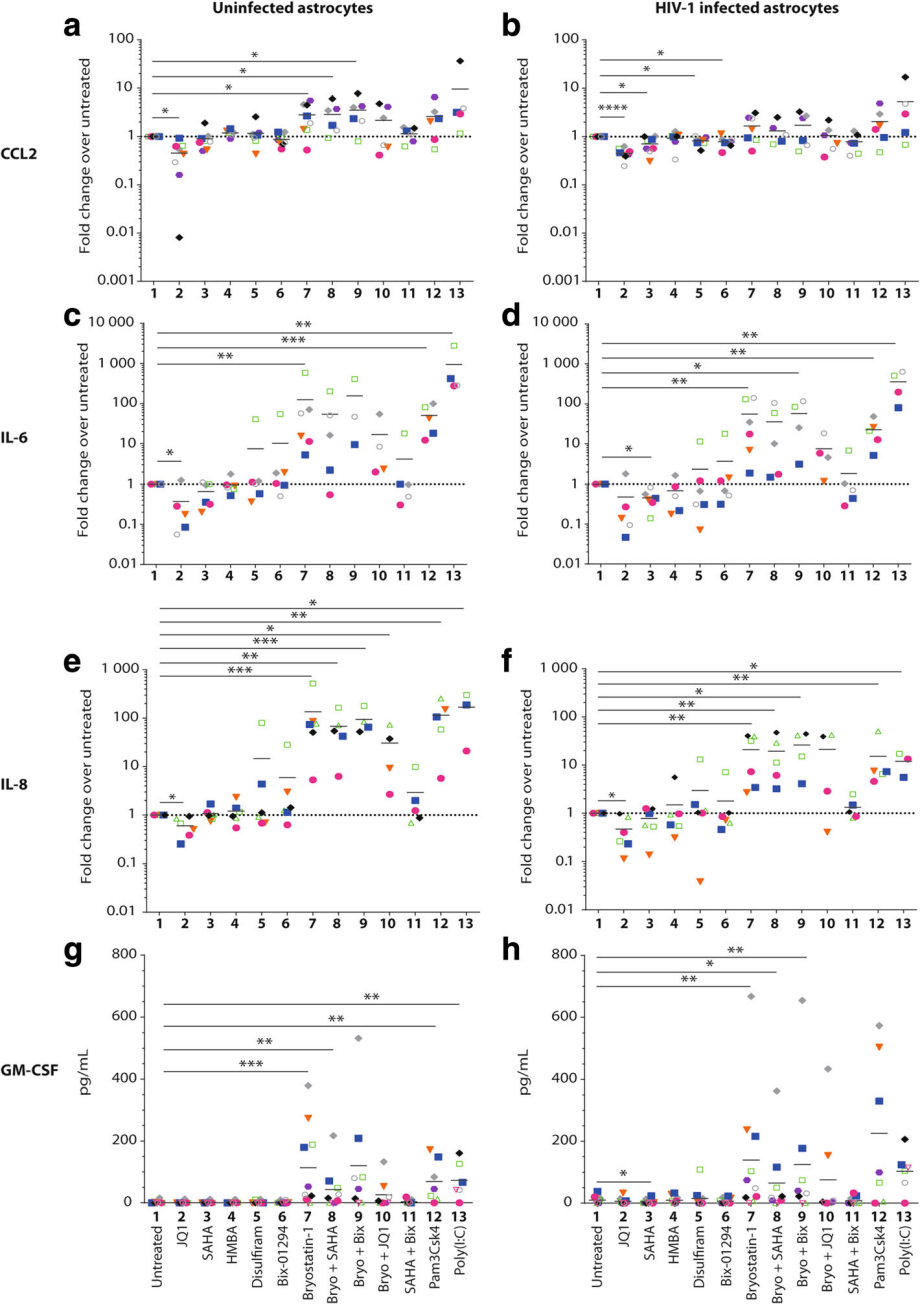
Differential modulation of chemoattractants and proinflammatory cytokines in astrocytes by bryostatin-1 and JQ1. Astrocytes were either left uninfected or infected with VSV-G-pseudotyped HIV-1 for 7 days and then treated for 24 h with the listed LRA. Cell-free supernatants were collected to measure **a**, **b** CCL2, **c**, **d** IL-6, **e**, **f** IL-8, and **g**, **h** GM-CSF production by commercial ELISA tests. Each value of uninfected (**a**, **c**, and **e**) and HIV-1-infected (**b**, **d**, and **f**) astrocytes either left untreated or treated with the listed LRA is presented in fold change over untreated cells except for the secretion of GM-CSF where results of **g** uninfected and **h** HIV-1-infected LRA-treated astrocytes are presented in raw data because values were under the detection threshold for untreated samples. Each colored dot represents a different donor, and the grand mean is shown for each experimental condition. Asterisks denote statistically significant data as defined by the Student *t* test (*P < 0.05, **P < 0.01, ***P < 0.001, and ****P < 0.0001)

Next, we evaluated the inflammatory effect of LRA on astrocytes by assessing secretion of various soluble factors. Supernatants were collected to measure CCL2, IL-6, IL-8, GM-CSF, CCL5, IL-1β, and TNF secretion by commercial ELISA tests. We monitored also TGFβ1 and interferon (IFN) α/β production using the reporting cell lines Mv1Lu and HEK-Blue™ IFNα/β, respectively. A representative astrocyte infection with VSV-G-pseudotyped eGFP-encoding HIV-1 particles is depicted in Fig. 1a. Virus infection leads to a modest increase in secretion of CCL2, IL-6, IL-8, and GM-CSF (Fig. 1b–e). However, production of IL-6, IL-8, and GM-CSF by astrocytes was augmented only for a subset of donors. Next, we assessed the effect of LRA, used either alone or in combination, on production of the listed soluble factors in both uninfected and virus-infected cells. Because of high donor-to-donor variability in cytokine secretion data, results are depicted as ratio over the untreated control sample for each donor. Secretion of TGFβ1, IL-1β, and TNF is neither modulated by HIV-1 nor by LRA treatment (data not shown). CCL5 production is increased by treatment with Bix-01294 (up to 4.3-fold) and bryostatin-1 when used alone or in combination (up to 3-fold) in uninfected astrocytes while IFNα/β production is induced only by JQ1 (up to 800 U/mL for uninfected and HIV-1-infected cells) (data not shown). Secretion of CCL2 (Fig. 2a, b) and proinflammatory cytokines IL-6 (Fig. 2c, d) and IL-8 (Fig. 2e, f) in response to LRA treatment exhibits similar secretion patterns although the LRA-mediated modulatory effect was more modest for CCL2. Indeed, while JQ1 treatment repressed secretion of CCL2, IL-6, and IL-8, bryostatin-1 induced a significant increase in production of CCL2 (up to 5.6-fold), IL-6 (up to 585-fold), and IL-8 (up to 518-fold). SAHA exerted a modest diminution of CCL-2, IL-6, and GM-CSF that reached statistical significance only in HIV-1-infected astrocytes. GM-CSF is produced by astrocytes only in response to bryostatin-1 (Fig. 2g, h). Treatment of cells with the listed combinations of LRA did not reveal any additive or synergistic activities, although interestingly enough, the bryostatin-1-mediated induction effect was generally dampened by the addition of JQ1. These results suggest that some LRA, especially bryostatin-1, induce a proinflammatory response in human astrocytes.

### Bryostatin-1 and JQ1 induce production of soluble factors by exerting an effect at the mRNA level

**Fig. 3:**
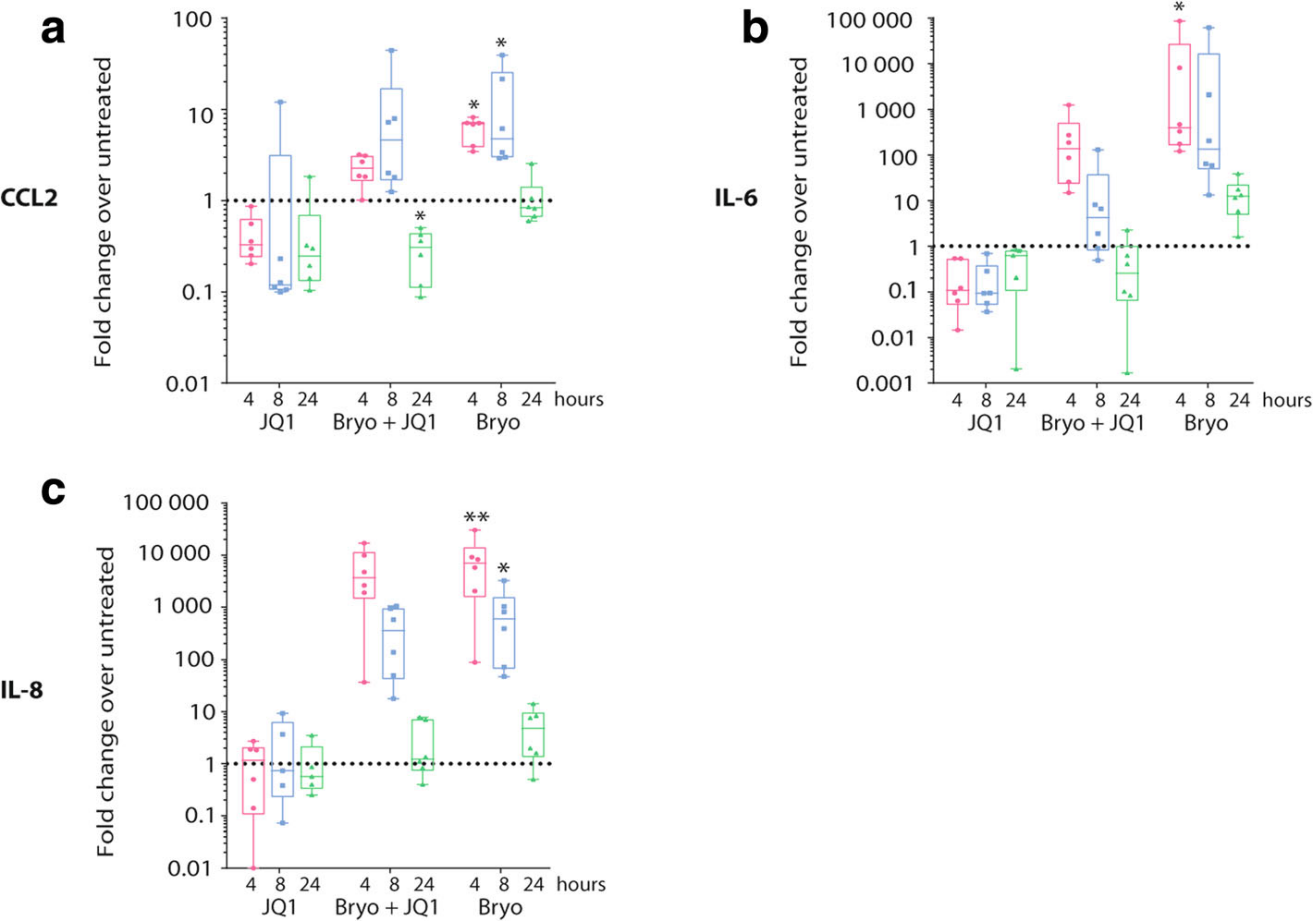
Bryostatin-1 and JQ1 affect CCL2, IL-6, and IL-8 gene expression. Astrocytes were treated with JQ1, bryostatin-1, or JQ1 and bryostatin-1 for 4, 8, or 24 h. Next, total RNA was extracted to measure mRNA expression of **a** CCL2, **b** IL-6, and **c** IL-8 by RT-qPCR. Data from six different donors are presented in fold change over the untreated condition. Asterisks denote statistically significant data as defined by the non-parametric Friedman test followed by Dunn’s multiple comparison test (*P < 0.05 and **P < 0.01)

Several studies have shown that the HIV-1 Tat-dependent upregulation of proinflammatory cytokines/chemokines occurs at the mRNA level through NF-κB- and/or AP-1-mediated signal transduction pathways [21]. Therefore, we tested whether the LRA-driven increase in secretion of CCL-2, IL-6, and IL-8 by astrocytes was similarly caused by alterations in gene expression. We previously demonstrated that bryostatin-1 induces a robust but transient increase in CCL2, IL-6, and IL-8 gene expression in the first 4–8 h of treatment in human BMVEC [9]. Consequently, we analyzed the effect of bryostatin-1 and JQ1 on CCL2, IL-6, and IL-8 gene expression after 4, 8, and 24 h of treatment in uninfected and HIV-1-infected astrocytes. Figure 3 shows that, as for BMVEC, bryostatin-1 mediates a strong upregulation of mRNA expression in the first 4 h of treatment, with a return to baseline levels after 24 h. The JQ1-driven diminution in gene expression was somewhat delayed but persisted up to 24 h, especially for CCL2. Interestingly, the combined action of bryostatin-1 and JQ1 resulted in an intermediate phenotype for CCL2 and IL-6 gene expression at the mRNA level. Similar mRNA expression profiles were obtained in HIV-1-infected astrocytes (data not shown).

### Bryostatin-1 modulates complement component 3 expression and glutamate uptake by astrocytes

Because astrocytes are the most abundant cell population in the brain, we also determined if LRA affect other basal activities that could lead to functional impairments of these cells. Oxidative stress plays a major role in a number of cellular damages leading to neurodegenerative disorders. We therefore studied generation of reactive oxygen species (ROS) by astrocytes in response to LRA using a commercial test as previously described [22]. Results suggest that astrocytes do not generate ROS in response to LRA (data not shown).

Reactive astrogliosis is a spectrum of changes in astrocytes that occur in response to various CNS insults and is promoted by various cytokines including IL-6 [23]. As IL-6 secretion is strongly increased by bryostatin-1, we studied the effect of LRA treatment on astrogliosis. We assessed by flow cytometry the intracellular glial fibrillary acidic protein (GFAP) expression, a common marker of astrogliosis, by astrocytes following either HIV-1 infection or treatment with LRA. Our results indicate that GFAP expression by astrocytes is neither affected by HIV-1 infection nor by a treatment with the tested LRA (data not shown).

**Fig. 4:**
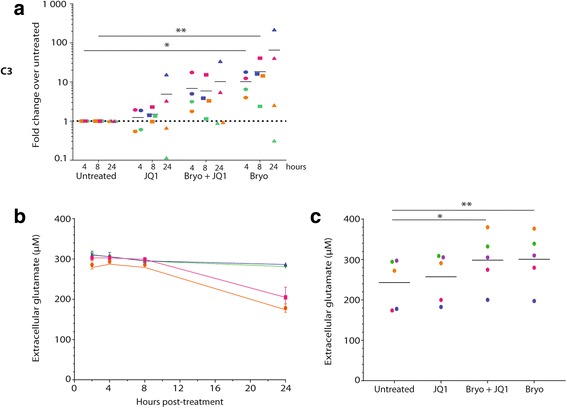
Bryostatin-1 modulates C3 mRNA expression and glutamate uptake by astrocytes. **a** Astrocytes were treated with JQ1, bryostatin-1, or JQ1 and bryostatin-1 for 4, 8, or 24 h. Total RNA was extracted to measure mRNA expression of C3 by RT-qPCR. Results from four different donors are presented in fold change over the untreated condition. **b**, **c** Astrocytes were either left untreated or treated with JQ1, bryostatin-1, or JQ1 and bryostatin-1. Glutamate levels in the supernatant were measured at 2, 4, 8, and 24 h post-treatment using a commercial glutamate assay kit. Data from one representative donor for the four time points (**b** orange: untreated, pink: JQ1, blue: bryostatin-1, green: JQ1 and bryostatin-1) and five donors for the 24 h treatment (**c**) are presented. Asterisks denote statistically significant data as defined by the non-parametric Friedman test followed by Dunn’s multiple comparison test (*P < 0.05 and **P < 0.01)

A recent study has suggested that GFAP might not be the ideal marker of astrocyte reactivity and proposed the complement component 3 (C3) as an indicator of astrogliosis [24]. We thus monitored C3 expression after treatment with JQ1, bryostatin-1, and JQ1 in combination with bryostatin-1. Results depicted in Fig. 4a show that bryostatin-1 induces a significant and sustained increase in C3 mRNA expression (average increase of 10.2-fold at 4 h, 18.4-fold at 8 h, and 65.6-fold at 24 h). On the contrary, JQ1 treatment alone does not affect C3 mRNA expression but appears to reduce the bryostatin-1-mediated induction when used in combination with bryostatin-1. Similar results were obtained when using HIV-1-infected astrocytes (data not shown),

Astrocytes are the main cells responsible for the maintenance of glutamate homeostasis in the brain through their uptake and release capacity. Thus, we investigated the overall effect of JQ1 and/or bryostatin-1 on exogenous glutamate uptake by mock- and HIV-1-infected astrocytes. Results show that while short treatments (up to 8 h) had no significant effect, a 24-h treatment with bryostatin-1 or the combined action of JQ1 and bryostatin-1 induced a significant decrease in astrocyte glutamate uptake (average decrease of 56% with bryostatin-1 and 54% with JQ1 and bryostatin-1) (Fig. 4b, c). JQ1 alone induced a modest decrease in glutamate uptake that did not reach statistical significance (Fig. 4c). Similar results were obtained with virus-infected astrocytes (data not shown).

### Recruitment of neutrophils through an in vitro BBB model is affected by astrocyte-conditioned medium after treatment with bryostatin-1 and JQ1

**Fig. 5:**
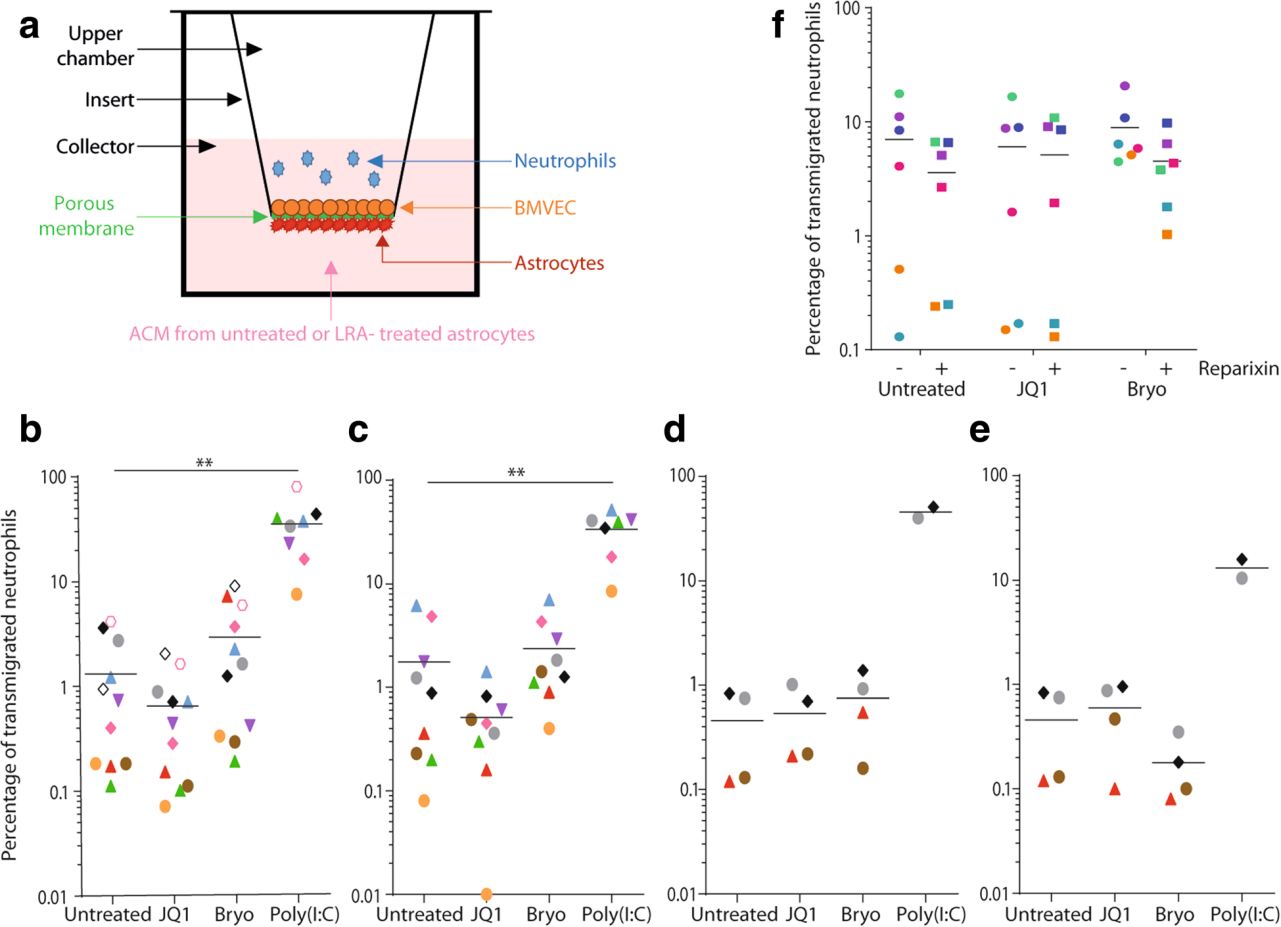
ACM from bryostatin-1- and JQ1-treated astrocytes affects neutrophil transmigration through an in vitro BBB model. **a** Schematic representation of the in vitro BBB model and neutrophil transmigration assay. Neutrophil transmigration was assessed using BBB containing **b**, **c** astrocytes and the human BMVEC line hCMEC/D3 or **d**, **e** hCMEC/D3 cells alone, in response to either **b**, **d** ACM from LRA-treated astrocytes or **c**, **e** LRA added in ACM from untreated astrocytes. **f** Neutrophils were pretreated with of reparixin (1 μM) for 45 min before performing the transmigration assay with ACM from LRA-treated astrocytes (circles: without reparixin: squares: with reparixin). The results are expressed in percentage of transmigrated neutrophils. Each colored dot represents a different sample. Asterisks denote statistically significant data as defined by the Student *t* test (*P < 0.05 and **P < 0.01)

It has been previously demonstrated that neutrophils are able to cross the BBB and could possibly be involved in the pathogenesis of some neurodegenerative and autoimmune diseases [25, 26]. Given that neutrophils are primarily recruited by the proinflammatory chemokine IL-8 and our data indicate that IL-8 secretion by astrocytes is affected by HIV-1 infection and treatment with bryostatin-1 and JQ1, we monitored the possible regulatory effect of the astrocyte secretory activity in response to bryostatin-1 and JQ1 on neutrophil transmigration through an in vitro human BBB experimental model (Fig. 5). We detected a comparable neutrophil recruitment when using ACM from uninfected and HIV-1-infected astrocytes (data not shown), thus suggesting that the modest virus-mediated increase in IL-8 secretion is insufficient by itself to influence neutrophil transmigration. However, ACM from JQ1-treated astrocytes diminishes neutrophil recruitment while ACM from bryostatin-1-treated astrocytes enhances transmigration of neutrophils through the BBB, although not to the same extent as the positive control consisting of ACM from poly(I:C)-treated astrocytes (Fig. 5b).

In order to differentiate the impact of LRA from that of the astrocyte secretome, we performed similar experiments with LRA supplemented with ACM from untreated astrocytes. We observed very similar patterns of neutrophil recruitment (Fig. 5c), thus suggesting a role not only of LRA-induced factors secreted by astrocytes but also of LRA themselves. However, it is possible that during the 4-h transmigration assay, LRA still present in ACM affect astrocytes that are part of the BBB model, thus inducing secretion of IL-8 and other chemoattractants. To shed light on this issue, we repeated these experiments with a BBB model composed of BMVEC only. A reduced neutrophil transmigration was measured, which confirms the importance of astrocyte-derived factors in leukocyte recruitment across the BBB (Fig. 5d, e). Given that both JQ1 and ACM from JQ1-treated astrocytes are not affecting neutrophil transmigration, it can be concluded that the negative effect of JQ1 was attributable to a direct drug action on astrocytes forming the BBB. Although a very modest increase in neutrophil recruitment is seen with ACM from bryostatin-1-treated astrocytes (Fig. 5d), their migration across the BBB was inhibited when the LRA was added back in ACM from untreated astrocytes (Fig. 5e). These data suggest that soluble factors secreted by astrocytes in response to bryostatin-1 tend to recruit neutrophils through a human BBB model, whereas bryostatin-1 by itself appears to repress this recruitment in the absence of astrocytes.

The small chemoattractant IL-8 is known to display distinct target specificity for the neutrophil. To evaluate the involvement of IL-8 secreted by astrocytes in response to LRA, we performed transmigration assays with neutrophils pretreated or not with reparixin, a noncompetitive allosteric inhibitor of IL-8 receptor activation. Reparixin had no significant impact on neutrophil recruitment by JQ1-treated ACM (Fig. 5f), therefore suggesting that the very low transmigration observed is induced by a chemokine other than IL-8. Surprisingly, although bryostatin-1-treated astrocytes secrete up to 200-fold more IL-8 that untreated astrocytes (Fig. 2e, f), reparixin pretreatment caused a similar 2-fold decrease in neutrophil transmigration in response to ACM either from untreated or bryostatin-1-treated astrocytes. These results suggest that either astrocytes secrete neutrophil chemoattractant(s) other than IL-8 or IL-8-mediated signaling is not completely inhibited by reparixin following a treatment with LRA. In summary, ACM from bryostatin-1-treated astrocytes cause a lower neutrophil recruitment despite the very high concentrations of IL-8 secreted by astrocytes in response to this LRA and a decrease in the number of transmigrated neutrophils is detected in response to bryostatin-1 compared to ACM from bryostatin-1-treated astrocytes. These seemingly contrasting observations suggest a deleterious effect of bryostatin-1 on neutrophil viability.

### Bryostatin-1 induces the formation of neutrophil extracellular traps

**Fig. 6:**
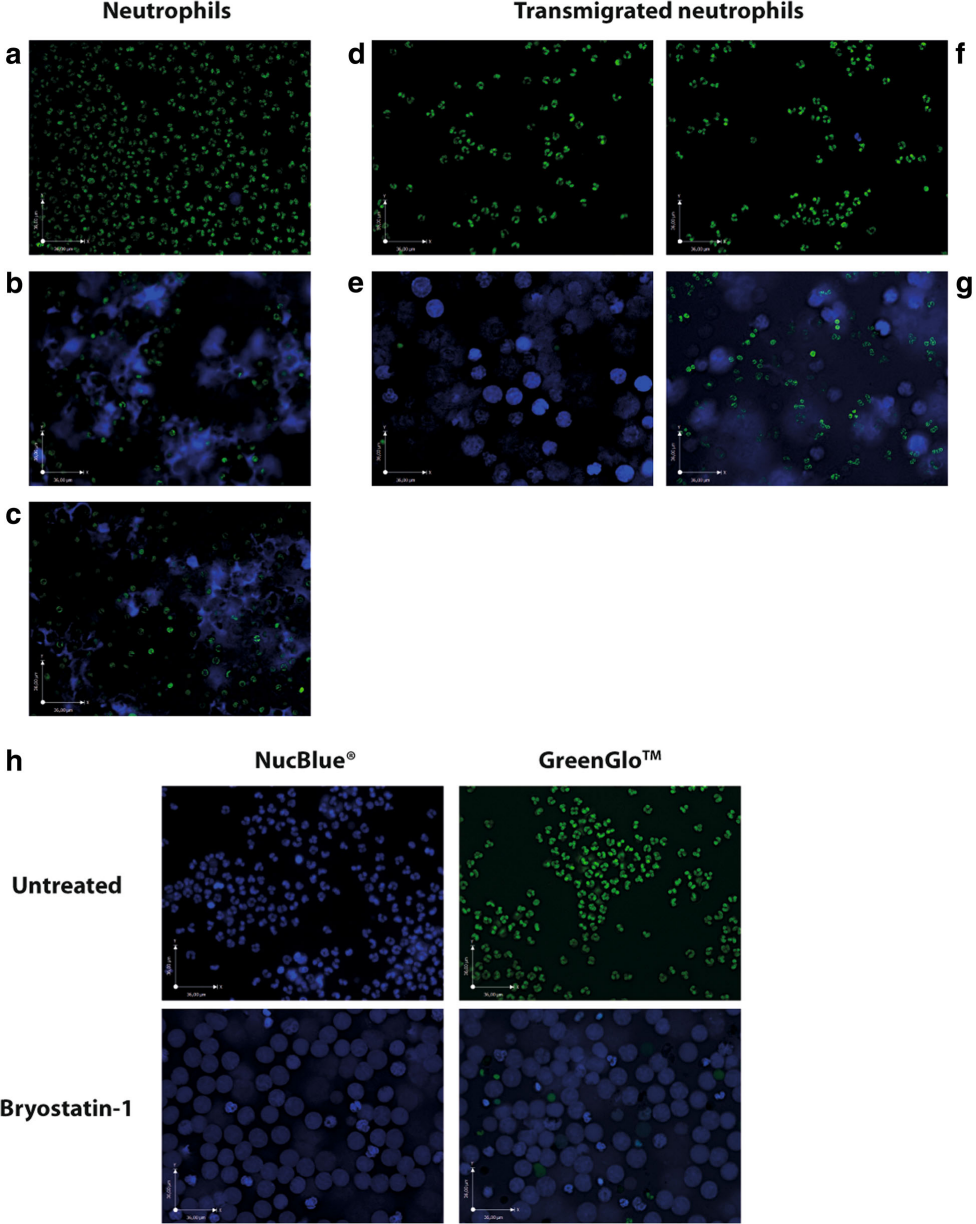
Bryostatin-1 induces NET formation following neutrophil recruitment across a BBB model. **a**–**c** Neutrophils were either left untreated (**a**) or treated with bryostatin-1 (25 nM) (**b**) or PMA (100 nM) (**c**) for 4 h. In parallel, neutrophils were allowed to transmigrate across a BBB model, with medium containing either IL-8 (1 nM) (**d**), bryostatin-1 (100 nM) (**e**), ACM from untreated astrocytes (**f**), or ACM from bryostatin-1-treated astrocytes (**g**), placed in the collector. After 4 h, inserts were discarded and DNA was labeled with 1× GreenGlo™ Safe DNA Dye. Cells were then imaged using both FITC (green) and UV (blue) filter sets. **h** Immunofluorescence microscopy validation of GreenGlo™ Safe DNA Dye labeling by comparison with the commonly used NucBlue® staining. Neutrophils were cultured for 4 h either in medium alone or with bryostatin-1 (25 nM). Next, DNA was labeled either with 1× GreenGlo™ Safe DNA Dye or with NucBlue® and imaged using both FITC and UV filter sets. Stained live cells were then visualized by inverted fluorescence microscope. Original magnification ×200. See also Additional file 2: Figure S2

In response not only to some invading pathogens but also to PKC activators such as phorbol 12-myristate 13-acetate (PMA), neutrophils externalize web-like chromatin strands decorated with antimicrobial peptides known as neutrophil extracellular traps (NETs) [27]. Because bryostatin-1 is a potent modulator of PKC, we next assessed if the observed reduction in transmigrated neutrophils in response to this compound might be related to NET formation. We stained neutrophils with the GreenGlo™ Safe DNA Dye, which is a dye that can be excited at both 295 and 490 nm. Surprisingly, nuclei from intact neutrophils were brighter when using a FITC filter set while extracellular DNA was best observed using a UV filter set (unpublished observations). Treatment of neutrophils with bryostatin-1 induced the formation of NET structures very similar to those induced by PMA (used as a positive control) (Fig. 6a–c). In addition, neutrophils migrating across the in vitro BBB model in response to ACM from untreated astrocytes (Fig. 6d) or culture medium supplemented with IL-8 to increase transmigration (Fig. 6f) did not display any extracellular DNA or cell damage, whereas ACM from bryostatin-1-treated astrocytes provoked a considerable NETosis among transmigrated neutrophils (Fig. 6e, g). In order to confirm the identity of the structures observed with this non-conventional staining, we labeled neutrophils either left untreated or treated with bryostatin-1 with NucBlue® (Hoechst® 33342 dye), a dye commonly used to label DNA. Similar extracellular DNA structures are detected with both stains. We reproduced these experiments with various concentrations of bryostatin-1, including the plasmatic concentration reported in vivo (i.e., 0.04 nM) [28]. Results indicate that brysostatin-1 causes a dose-dependent induction of NET formation. NETosis can be observed at a concentration as low as 0.4 nM, and doses higher than 1 nM induced NET formation in most neutrophils (Additional file 2: Figure S2). These results suggest that bryostatin-1 might promote recruitment of neutrophils in the CNS followed by suicidal NETosis.

## Discussion

The use of LRA to reactivate HIV-1 from latently infected cells has been proposed as part of the “shock and kill” therapeutic strategy, which is aimed at achieving a sterilizing cure [2]. Although many reports have described the effectiveness of various LRA in reactivating the latent virus in CD4^+^ T cells [3], the possible eradication of brain reservoirs with this strategy is still undefined. To our knowledge, only one study has assessed the impact of LRA on some functions of CNS cell types such as BMVEC [9] and the present work is extending these investigations to astrocytes, the most abundant cell type in the brain.

Our initial findings demonstrate that certain basic functions of astrocytes are not affected by LRA. Indeed, LRA do not disturb astrocyte metabolic activity and do not trigger ROS production. However, we report that bryostatin-1, when used either alone or in combination with other LRA, triggers secretion by astrocytes of several chemokines and proinflammatory cytokines such as CCL2, IL-6, IL-8, and GM-CSF. The LRA-mediated effect on production of such soluble factors is seen in both uninfected and HIV-1-infected astrocytes and is exerting its effect at the mRNA level. This observation is of high clinical significance given that a very low percentage of HIV-1-infected astrocytes is detected under in vivo conditions. Bryostatin-1 is a PKC modulator that has been recently tested in a clinical trial for HIV-1 reactivation [28]. Plasma concentrations reported by Gutierrez and colleagues after a 20 μg/m^2^ single-dose administration were 100-fold lower than bryostatin-1 concentrations used in previously described in vitro studies including the current one [9, 29–31]. However, Gutierrez and co-workers did not detect any HIV-1 reactivation at this very low plasma concentration. Thus, higher doses of bryostatin-1 will be necessary to reactivate the virus in vivo and the adverse effects described in our work might be reached under such conditions. Indeed, as previously mentioned, astrocytes release CCL2, IL-6, IL-8, and GM-CSF in response to a treatment with bryostatin-1. All these soluble factors can exhibit neuroprotective effects in the CNS during some trauma or diseases. CCL2, by reducing glutamate levels increased by *N*-methyl-d-aspartate (NMDA) and Tat protein, and IL-6, by preventing ROS and Ca^2+^ excitotoxicity in Parkinson’s and Huntington’s diseases, exert neuroprotective properties [32, 33]. GM-CSF participates in the neuronal repair after a traumatic injury to the CNS [34] and IL-8 may increase neuronal survival after a traumatic brain injury by promoting the production of nerve growth factor (NGF) by astrocytes [35]. However, it has also been shown that a CCL2 exposure of murine astrocytes and BMVEC induced a structural change of the actin cytoskeleton and a redistribution of tight junction proteins leading to a more porous BBB and a facilitation of leukocyte migration into the CNS [36]. Moreover, Ehrhart and colleagues showed significant increases in levels of IL-8 in the blood from amyotrophic lateral sclerosis (ALS) patients compared to controls, these higher levels of IL-8 being correlated with disease progression [37]. It has also been demonstrated that IL-6 levels are elevated in the CSF of patients with chronic schizophrenia. Moreover, IL-6 is able to activate the kynurenine pathway leading to an increase of the kynurenic acid production, a compound that has been consistently reported at elevated levels in the CSF or in the post-mortem brain of patients with schizophrenia [38]. Finally, McQualter and co-workers have detected the formation and expansion of inflammatory lesions within the CNS during experimental autoimmune encephalomyelitis, a mouse model for human multiple sclerosis (MS) that is highly dependent on GM-CSF [39]. All these studies and many others have established or suggested detrimental roles in various brain disorders and their progression (MS, Alzheimer disease (AD), cerebral stroke, ALS, and more) and BBB permeability for these four cytokines that are all upregulated by bryostatin-1 in astrocytes [32, 33, 40, 41].

Astrogliosis is an astrocyte response to neuronal insults due to injury or disease. Although constitutive astrogliosis exerts beneficial functions for the brain such as restriction of CNS inflammation, neuronal protection, BBB repair, and wound closure, this process can also lead to harmful effects such as exacerbation of inflammation or interference with synapse sprouting or axonal growth [42]. Human astrogliosis is commonly assessed in vivo by an increase of GFAP expression, which was never shown in vitro. Thus, even if we did not observe any increase in GFAP expression after LRA treatments, bryostatin-1 might induce astrogliosis. Indeed, it has been suggested that (i) bryostatin-1 activates latent HIV-1 through a PKC- and NF-κB-dependent mechanism [30] and (ii) reactive astrocytes might be induced by NF-κB signaling [24]. It is thus possible that bryostatin-1 induces the so-called A1 neuroinflammatory reactive astrocytes. Our results showing a bryostatin-1-mediated increase in C3 expression confirm this postulate as C3 is specifically upregulated in inflammatory reactive astrocytes and has therefore been proposed as a marker of astrogliosis [24].

It is well known that a tight regulation of brain glutamate concentrations is vital, as elevated levels of extracellular glutamate may lead to excitotoxicity [43]. We demonstrate here that the glutamate concentrations are significantly higher in supernatants from bryostatin-1-treated astrocytes compared to untreated cells. Considering that astrocytes maintain glutamate homeostasis by a delicate balance between uptake and release processes, it can be proposed that bryostatin-1 may disrupt this equilibrium by either decreasing glutamate uptake and/or increasing its release by astrocytes. Excitatory amino acid transporters 1 and 2 (EAAT1/GLAST and EAAT2/GLT-1, respectively) expressed at the astrocyte plasma membrane are responsible for extracellular glutamate uptake by astrocytes. Their cellular localization is controlled by PKC, and it has been shown that PKC activation drives internalization of EAAT1/2 to endosomal compartments [44]. As bryostatin-1 is a PKC activator, the observed increase in extracellular glutamate concentration could be caused by internalization of the transporter. Compared to glutamate uptake, astrocytic glutamate release is far more complex and can occur by numerous mechanisms [45] in which the precise bryostatin-1- or PKC-dependent modulatory effects are still unknown. If an occasional use of bryostatin-1 should not lead to a chronic excitotoxicity, it could induce an acute excitotoxicity, which is mainly mediated by an increase of extracellular glutamate levels [43].

We also demonstrate that ACM from bryostatin-1-treated astrocytes causes a significant transmigration of neutrophils through an in vitro BBB model. It is possible that neutrophil transmigration is facilitated by a bryostatin-1-mediated disruption of the BBB as we previously described [9] and the elevated production of IL-8 by astrocytes in response to the LRA. Neutrophils are essential for protective immunity during infection and tissue repair. However, by their release of antimicrobial proteins, proteases, and oxidants during the inflammatory response, recruited neutrophils may also cause severe side effects in the brain. For example, when activated in the inflammation site, neutrophils release CCL20 that mediates recruitment of Th17 cells [46]. In turn, Th17 cells, along with migrating neutrophils, produce Il-17, which is toxic to neurons, induces BBB breakdown by decreasing the expression of tight junction proteins, promotes monocyte infiltration into the CNS via an ICAM-1-dependent mechanism, and activates glial cells that could release mediators contributing to brain damage [26, 47]. Furthermore, our data show that apart from inducing neutrophil transmigration across the BBB, bryostatin-1 induces NET formation in the recruited neutrophils. Since bryostatin-1 is a PKC activator similar to PMA, a well-known NETosis inducer, this observation is not surprising. NETosis is a defense mechanism by which neutrophils eliminate pathogens by releasing their chromosomal DNA, histones, and granule contents to the extracellular space [27]. It has been shown that NETs act in the antiviral response against HIV-1 through virus capture and neutralization via production of α-defensin and myeloperoxidase, and would be therefore a potential mechanism to protect against HIV-1 [48]. Although there is a paucity of data about the formation of NETs in humans, it has nonetheless been suggested that neutrophils and NETs may play a role in AD pathology as they have been observed close to the amyloid β plaques in the CNS of AD patients [26]. Moreover, components released during NETosis contribute to the loss of BBB integrity [49], and NET formation may play a role in systemic lupus erythematosus through the induction of type-I IFN production [50].

Bryostatin-1 is actually in a phase II clinical trial (NCT02431468) to assess its potential for the treatment of moderately severe to severe stages of AD. However, we estimated that the 40-μg dose administrated in this trial is similar to the 20 μg/m^2^ dose used in the clinical trial conducted by Gutierrez and his group. Thus, this low concentration of bryostatin-1 may be safe and efficient enough for the treatment of AD, but as specified above, this dose would not be sufficient per se to reactivate latent HIV-1 and higher doses could lead to the adverse effects described in the present work.

Interestingly, JQ1 presents quite different outcomes than bryostatin-1. For instance, JQ1 inhibits secretion of multiple soluble factors (i.e., CCL2, IL-6, IL-8, and GM-CSF) by astrocytes and suppresses neutrophil recruitment across an in vitro BBB while it does not induce astrogliosis and has no effect whatsoever on glutamate uptake/release and NETosis. JQ1 is also the only LRA tested, when used alone or in combination with bryostatin-1, that induces production of IFNα/β by astrocytes (data not shown). JQ1 is a small-molecule inhibitor of BET bromodomain (BRD) protein binding that has been shown to abolish inflammation, endothelium activation, and leukocyte transmigration [51, 52]. It has been reported that, under inflammatory stimuli, the transcription factor NF-κB recruits the member of BET family BRD4 [52]. Both NF-κB and BRD4 direct the formation of dynamic super-enhancers that control transcription of genes driving the inflammatory response. By inhibiting BRD4, JQ1 prevents formation of those super-enhancers and acts as an anti-inflammatory molecule. Moreover, a pretreatment with JQ1 inhibits the phenotypic features of BMVEC proinflammatory activation by reducing leukocyte rolling and neutrophil transmigration both in vivo and in vitro using EC monolayers [52]. In our hands, we noticed a decrease in the number of transmigrated neutrophils in response to ACM from JQ1-treated astrocytes in an in vitro BBB experimental model made of BMVEC and astrocytes (Fig. 5b, c) but not with a BBB containing only BMVEC (Fig. 5d, e). This suggests that the inhibitory activity of JQ1 on neutrophil transmigration through the BBB is mainly due to a modulatory effect on astrocyte-mediated secretion of chemokines in response to JQ1.

## Conclusions

Altogether, our data suggest that JQ1 might dampen the proinflammatory activity of bryostatin-1, except for glutamate uptake/release by astrocytes. Other LRA such as SAHA and BIX-01294 did not display any deleterious effect on astrocytes; however, when used in combination with bryostatin-1, they did not attenuate its overall effect. Therefore, our data suggest that JQ1 can be considered as a valuable LRA to use in combination with bryostatin-1 in order to reduce a potential harmful inflammation in the brain. In summary, our study provides evidence that the safety of the “shock and kill” approach needs to be further investigated in light of the distinctive characteristics of the CNS.

## Additional files

Additional file 1: Figure S1. LRA are well-tolerated by astrocytes. The metabolic activity of astrocytes was assessed using a MTS assay at 7 days following infection with VSV-G-pseudotyped HIV-1 and 24 h of treatment with the listed LRA. (A) The overall modulatory effect of HIV-1 infection alone on metabolic activity is presented in raw data (absorbance at 490 nm). Each value of uninfected (B) and HIV-1-infected (C) astrocytes either left untreated or treated with LRA are presented in percentage of the untreated condition. Each colored dot represents a different donor sample, and the grand mean is shown as a horizontal line. Asterisks denote statistically significant data as defined by the Student *t* test (*P < 0.05). (TIFF 238 kb)

Additional file 2: Figure S2. Bryostatin-1 does not trigger NETosis up to a concentration of 0.2 nM. NET formation after neutrophils were treated for 4 h with the indicated concentrations of bryostatin-1 was assessed by microscopy using the 1× GreenGlo™ Safe DNA Dye to label nuclei and NET DNA. (TIFF 2395 kb)

## Abbreviations

ACM: Astrocyte-conditioned medium
AD: Alzheimer disease
ALS: Amyotrophic lateral sclerosis
BBB: Blood-brain barrier
Bix-01294: 2-(Hexahydro-4-methyl-1*H*-1,4-diazepin-1-yl)-6,7-dimethoxy-*N*-[1-(phenylmethyl)-4-piperidinyl]-4-quinazolinamine trihydrochloride hydrate
BMVEC: Brain microvascular endothelial cells
BRD: BET bromodomain
C3: Complement component 3
cART: Combined antiretroviral therapy
CCL(x): Chemokine (C-C) ligand
CNS: Central nervous system
EAAT(x): Excitatory amino acid transporter
GFAP: Glial fibrillary acidic protein
GM-CSF: Granulocyte-macrophage colony-stimulating factor
HAND: HIV-1-associated neurocognitive disorders
HDAC: Histone deacetylase
HMBA: *N*,*N*’-Hexamethylene bis(acetamide)
HMT: Histone methyltransferase
IFN: Interferon
IL-(x): Interleukin
LRA: Latency-reversing agents
MCP-1: Monocyte chemoattractant protein-1
MLEC: Mink lung epithelial cells
MS: Multiple sclerosis
NETs: Neutrophil extracellular traps
NGF: Nerve growth factor
NMDA: *N*-methyl-d-aspartate,
PKC: Protein kinase C
PMA: Phorbol 12-myristate 13-acetate
poly(I:C): Polyinosinic/polycytidylic acid
ROS: Reactive oxygen species
SAHA: Suberoylanilide hydroxamic acid
TGFβ: Transforming growth factor beta
TLR: Toll-like receptor
