# Supplementary figures and images for: Contrasting effect of the latency-reversing agents bryostatin-1 and JQ1 on astrocyte-mediated neuroinflammation and brain neutrophil invasion
Source: J Neuroinflammation. 2017 Dec 11;14:242. doi: 10.1186/s12974-017-1019-y (PMC5725742; doi:10.1186/s12974-017-1019-y)

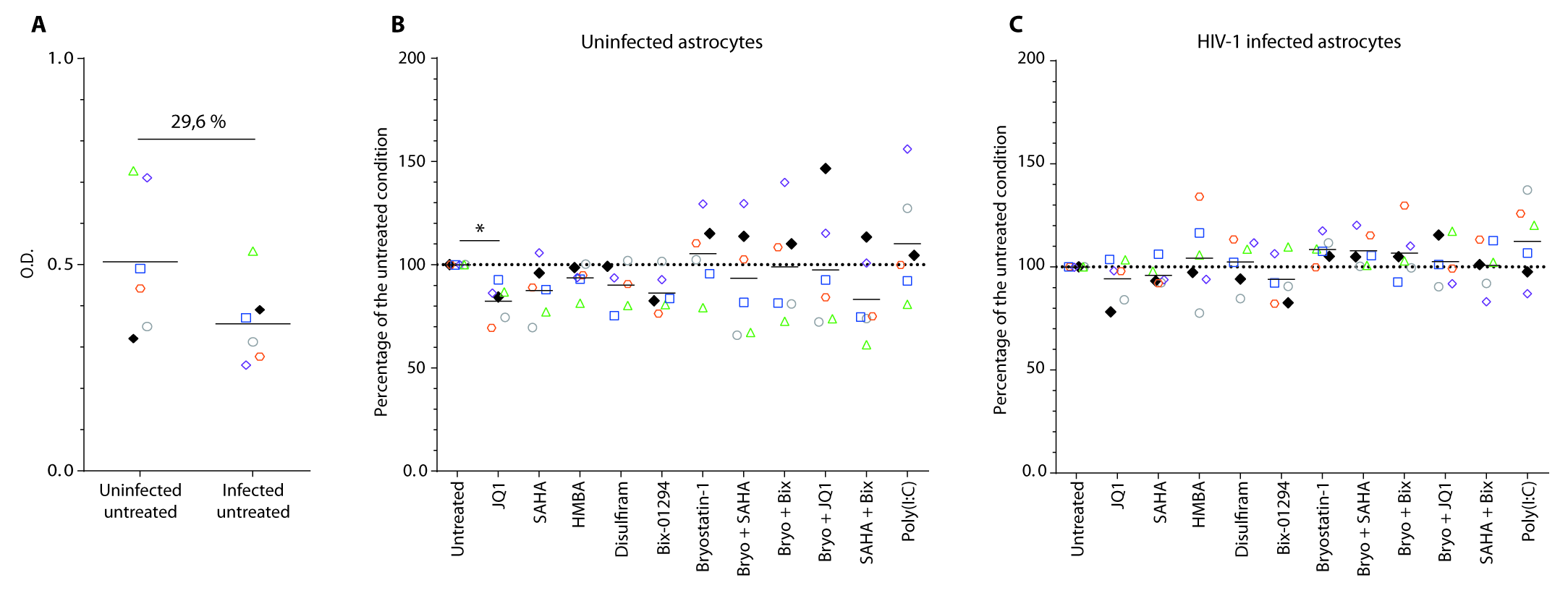

Supplement: Supplementary file 1 — LRA are well-tolerated by astrocytes. The metabolic activity of astrocytes was assessed using a MTS assay at 7 days following infection with VSV-G-pseudotyped HIV-1 and 24 h of treatment with the listed LRA. (A) The overall modulatory effect of HIV-1 infection alone on metabolic activity is presented in raw data (absorbance at 490 nm). Each value of uninfected (B) and HIV-1-infected (C) astrocytes either left untreated or treated with LRA are presented in percentage of the untreated condition. Each colored dot represents a different donor sample, and the grand mean is shown as a horizontal line. Asterisks denote statistically significant data as defined by the Student t test (*P < 0.05). (TIFF 238 kb) [file 12974_2017_1019_MOESM1_ESM.tif]

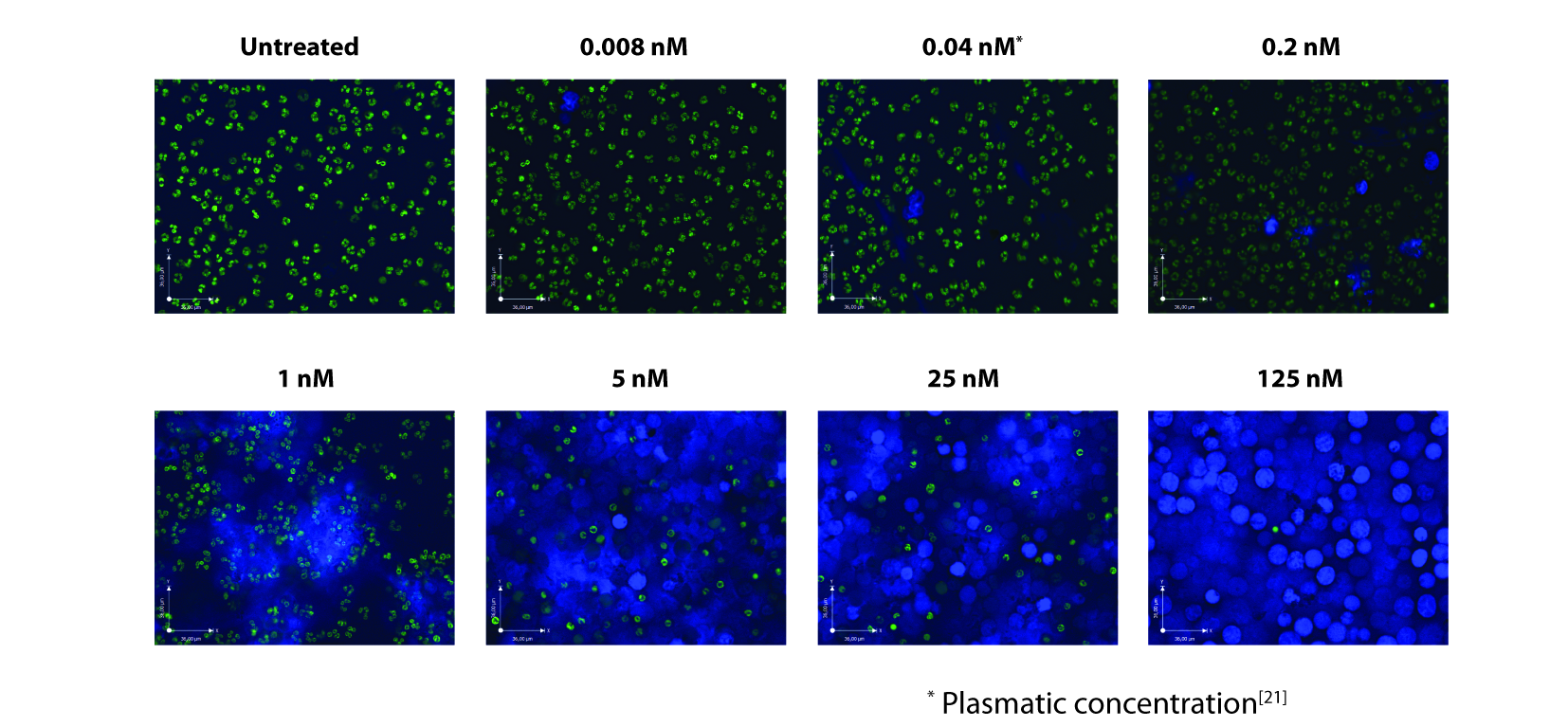

Supplement: Supplementary file 2 — Bryostatin-1 does not trigger NETosis up to a concentration of 0.2 nM. NET formation after neutrophils were treated for 4 h with the indicated concentrations of bryostatin-1 was assessed by microscopy using the 1× GreenGlo™ Safe DNA Dye to label nuclei and NET DNA. (TIFF 2395 kb) [file 12974_2017_1019_MOESM2_ESM.tif]
